# Postoperative enteral nutrition timing and risk variation in elderly patients with perforated peptic ulcer

**DOI:** 10.3389/fnut.2026.1781389

**Published:** 2026-07-24

**Authors:** Cang-Dian Huang, Jia-Lin Chen, Bao-Wei Xu, Shuai Chen, Peng-Sheng Tu, Jun-Rong Zhang, Xian-Qiang Chen

**Affiliations:** Department of Emergency Surgery, Fujian Medical University Union Hospital, Fuzhou, Fujian Province, China

**Keywords:** early enteral nutrition, elderly perforated peptic ulcers, machine learning, optimal time, prognoses

## Abstract

**Purpose:**

Enteral nutrition (EN) is important for postoperative recovery in elderly patients with perforated peptic ulcer (ePPUs), but the optimal initiation timing remains unclear. This study aimed to analyze the association between EN timing and adverse outcome in ePPUs.

**Methods:**

This retrospective cohort study enrolled 142 ePPUs who underwent surgical repair between May 2017 and May 2025. Postoperative outcomes were stratified using a two-step clustering (TSC) model. Subsequently, machine learning was further applied to evaluate how the risk of adverse outcomes varied with delayed enteral nutrition initiation.

**Results:**

Among 142 patients, 104 were classified into the NAOG and 38 into the AOG. Compared with the NAOG, the AOG showed higher rates of intra-abdominal infection (86.8% vs. 0.0%, *p* < 0.001) and anastomotic leakage (23.7% vs. 0.0%, *p* < 0.001), longer hospital stay (22.50 [15.00–34.50] vs. 9.50 [8.00–12.25] days, *p* < 0.001) and ICU stay (106.50 [0.00–308.00] vs. 0.00 [0.00–0.00] hours, *p* < 0.001). Meanwhile, the AOG had higher preoperative serum creatinine levels (130.50 [85.00–192.50] vs. 83.50 [64.00–107.00] μmol/L, *p* < 0.001) and larger perforation sizes (10.00 [7.25–15.00] vs. 8.00 [5.00–10.00] mm, *p* < 0.001). In the primary DML analysis, each 1-day delay in postoperative EN initiation was associated with a 3.55% (95% CI, 1.41–6.61%) increase in adverse outcome risk. Postoperative day 6 corresponded both to the point at which the spline-based risk residual returned to zero and to the cutoff with the highest AUC in the decision-tree analysis (AUC = 0.724).

**Conclusion:**

Delayed postoperative EN initiation was associated with a cumulative increase in adverse outcome risk, which appeared to shift to a higher-risk level after approximately postoperative day 6.

## 1 Introduction

Perforated peptic ulcer (PPU) is characterized by a full-thickness defect of the gastric or duodenal wall that permits gastrointestinal contents to spill into the peritoneal cavity, rapidly leading to acute peritonitis (1–3). Clinically, it typically presents with sudden and severe abdominal pain accompanied by signs of an acute abdomen (4). PPU develops in approximately 2–10% of patients with peptic ulcer disease and carries a mortality rate of 4–20%, making it one of the most common surgical emergencies worldwide (3, 5). With the continuing trend of global population aging, the demographic profile of emergency surgical patients is shifting toward older cohorts (6, 7). Elderly perforated peptic ulcer (ePPUs) are particularly vulnerable: atypical symptoms frequently delay diagnosis, whereas multiple comorbidities, malnutrition, and diminished physiological reserve markedly increase operative risk (8, 9). In this population, complication rates have been reported at 40–50%, with mortality exceeding 20% (6, 10, 11). Given these risk factors, greater attention should be directed toward the perioperative management of ePPUs.

Enteral nutrition (EN), defined as the oral or gastrointestinal tube delivery of nutritional support through the gastrointestinal tract, is a key component of perioperative care in gastrointestinal surgery (12). As a cornerstone of enhanced recovery after surgery (ERAS), early EN has consistently been shown to preserve gut barrier function, reduce infectious complications, and promote postoperative recovery, thereby improving clinical outcomes (13–15). Malnutrition occurs in nearly 80% ePPUs and may further compromise postoperative recovery by limiting nutritional reserves (7, 16). Although extensive evidence and clinical guidelines have demonstrated that early EN does not increase the risk of anastomotic leakage, its application in ePPUs still remained debatable (17, 18). For ePPUs, severe inflammation, extensive contamination around the perforation site, limited healing capacity, and insufficient nutritional reserves further impede postoperative recovery (9, 11, 16). Balancing the risk of anastomotic leakage against the benefits of early EN has long been a clinical dilemma for surgeons (14). Current guidelines consistently advocate early EN as part of postoperative care (12); however, in ePPUs undergoing perforation repair, the decision regarding the initiation of EN still largely depends on subjective clinical judgment and lacks robust objective evidence. To address this gap, we applied machine learning (ML) methods to assess the relationship between EN timing and postoperative outcomes.

ML has demonstrated substantial potential in gastrointestinal surgery due to its capacity to process high-dimensional, multivariate, and nonlinear clinical data (19–21). It has shown promise in postoperative risk stratification, prediction of anastomotic leakage, and analysis of complex clinical decision-making among patients with acute abdominal conditions. DML, an emerging causal inference technique, has been increasingly applied in medicine to estimate treatment effects in clinical settings and may help reduce confounding bias in high-dimensional datasets (22–24).

In this study, we applied multiple ML-based approaches to investigate the association between delayed EN initiation and postoperative outcomes in ePPUs, with the aim of informing clinical decision-making regarding EN timing.

## 2 Materials and methods

### 2.1 Study design and population

This single-center retrospective cohort study included 142 elderly patients with primary gastric or duodenal perforation who were admitted to the Department of Emergency Surgery, Union Hospital, Fujian Medical University, between May 2017 and May 2025. All operations were performed as damage-control-oriented surgical repairs rather than gastric or duodenal resections. The inclusion criteria were as follows: (1) primary gastric or duodenal perforation confirmed intraoperatively; and (2) age ≥60 years. The exclusion criteria were as follows: iatrogenic perforation (*n* = 3), anastomotic leakage-related perforation (*n* = 2), and perforation after neoadjuvant therapy for gastric cancer (*n* = 9).

### 2.2 Collection of clinical variables

Clinical data were extracted from the hospital electronic medical record system. Baseline variables included age, sex, symptom duration, signs of peritonitis signs, abdominal surgery history, comorbidities, and smoking or alcohol history. Preoperative physiological and laboratory indicators covered systolic blood pressure, temperature, heart rate, ASA class (I–II vs. III–V), hemoglobin, platelets, albumin, electrolytes, white blood cell count, neutrophil and lymphocyte counts, liver enzymes, creatinine, glucose, and coagulation markers. Imaging variables included multifocal free intraperitoneal gas, defined as extraluminal air in ≥2 peritoneal or retroperitoneal compartments or clustered bubbles within a single compartment (25), and extraperforation bowel wall thickening, defined as mural thickening beyond the segment adjacent to the perforation (26). Intraoperative findings included perforation size and site, intra-abdominal effusion >1,000 mL, and bacterial culture results.

Postoperative outcomes included intra-abdominal infection (IAI), anastomotic leakage (AL), pre-discharge hemoglobin and albumin levels, intensive care unit (ICU) stay, and length of hospital stay (LOS). pIAI was defined as ongoing or recurrent intra-abdominal sepsis requiring intervention, characterized by purulent or turbid drainage, clinical signs of infection, or imaging-confirmed abscess requiring continuous or negative-pressure irrigation (27–29). AL was defined as leakage at the repair site and diagnosed based on postoperative fever, abdominal pain or distension, abnormal drainage containing gastrointestinal contents, and computed tomography (CT) findings of a perirepair abscess or fluid collection (4, 30).

### 2.3 Prognostic phenotyping

To comprehensively evaluate postoperative recovery, two-step clustering (TSC) was performed using IAI, AL, LOS, ICU stay, and pre-discharge hemoglobin and albumin levels. Patients were classified into the adverse outcome group (AOG) and the non-adverse outcome group (NAOG), representing poorer and more favorable overall postoperative profiles, respectively. This TSC-derived composite phenotype, rather than any single complication, was used as the outcome variable in subsequent analyses. Differences in the included clustering variables between AOG and NAOG were further compared to assess the discriminative performance of the classification, and the relative contribution of each variable was evaluated using standardized weight coefficients.

### 2.4 DML-based effect estimation and nonlinear threshold analysis

Using the TSC-derived phenotype as the outcome, preoperative candidate variables were first tested by univariate analysis, followed by LASSO selection. The treatment variable was postoperative EN initiation timing, and the outcome variable was the TSC-derived prognostic phenotype (AOG vs. NAOG). The retained variables were then included in the subsequent DML model for adjustment. By separately modeling the treatment and outcome processes, DML estimates the conditional treatment and outcome expectations while reducing regularization bias. In the primary analysis, the treatment model was specified as linear regression (LR), and the outcome model was fixed as logistic regression. To test the robustness of the treatment-model specification, additional learners were evaluated while keeping the outcome model unchanged, including random forest (RF), support vector machine (SVM), multilayer perceptron (MLP), and extreme gradient boosting (XGB). Comparisons focused on the estimated average treatment effect (ATE), the standard deviation of the ATE across five-fold cross-fitting, and the direction of the effect.

Based on the primary DML model, a five-knot B-spline regression was fitted to the DML-residualized outcome to examine the potential nonlinear association between postoperative EN initiation timing and adverse outcome risk. Using the same modeling framework, a postoperative date-based day-by-day scan with a single-level decision tree was performed to assess the discriminatory performance of each postoperative day as a candidate threshold and identify clinically potentially meaningful time cutoffs.

### 2.5 Statistical analysis

For variables with a missing rate of less than 10%, missing values were imputed using a random forest–based method. The Kolmogorov–Smirnov test was used to assess the normality of continuous variables. Normally distributed variables were expressed as mean ± standard deviation (SD) and compared using the independent-samples t test, whereas non-normally distributed variables were presented as median (interquartile range, IQR) and compared using the Mann–Whitney U test. Categorical variables were summarized as counts (percentages) and compared using the χ^2^ test. All tests were two-sided, and *p* < 0.05 was considered statistically significant. Except for the TSC analysis, which was performed in SPSS version 27.0, all other analyses were conducted in a Python 3.9.

### 2.6 Ethics approval

This study was approved by the Ethics Committee of Fujian Medical University Union Hospital (Approval No. 2026KY730). The requirement for informed consent was waived owing to the retrospective nature of the study, in accordance with national regulations. All procedures were performed in compliance with the ethical standards of the institutional and/or national research committee and with the 1964 Declaration of Helsinki and its later amendments.

## 3 Results

### 3.1 Clustering of prognostic phenotypes and comparison of clinical characteristics

**Figure 1:**
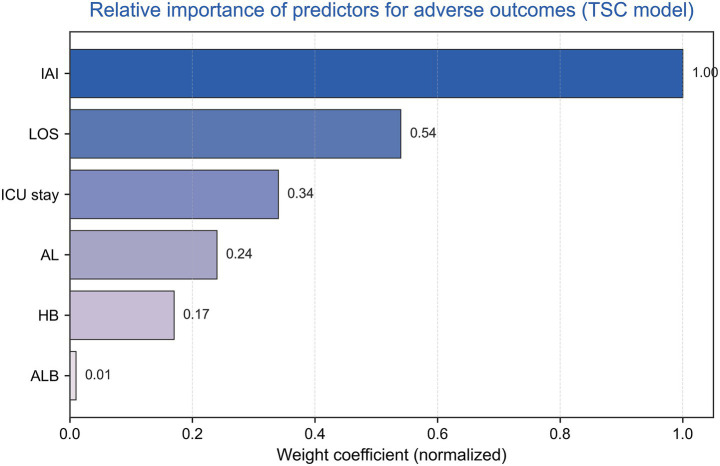
Relative importance of postoperative outcome indicators in the two-step clustering (TSC) model. TSC, two-step clustering; IAI, intra-abdominal infection; LOS, length of hospital stay; ICU, intensive care unit; AL, anastomotic leakage; HB, hemoglobin; ALB, albumin.

A clustering model was constructed based on multiple postoperative indicators following TSC, including a total of 142 patients, among whom 104 were classified into NAOG and 38 into AOG. In the clustering model, variables ranked by descending weight coefficients were as follows (Table 1; Figure 1): IAI(weight = 1.00; AOG 86.8% vs. NAOG 0.0%; *p* < 0.001), LOS (0.54; 22.50 [15.00–34.50] vs. 9.50 [8.00–12.25]; *p* < 0.001), ICU stay (0.34; 106.50 [0.00–308.00] vs. 0.00 [0.00–0.00]; *p* < 0.001), anastomotic leakage (0.24, 23.7% vs. 0.0%; p < 0.001), hemoglobin level (0.17, 94.83 ± 17.09 vs. 110.28 ± 19.54; *p* < 0.001), and albumin level (0.01, 32.36 ± 7.07 vs. 32.99 ± 4.15; *p* = 0.512).

**Table 1:** Postoperative outcomes by TSC grouping.

Variable	NAOG (*n* = 104)	AOG (*n* = 38)	*p*-value
IAI, n (%)			<0.001
No	104 (100.0)	5 (13.2)	
Yes	0 (0.0)	33 (86.8)	
AL, n (%)			<0.001
No	104 (100.0)	29 (76.3)	
Yes	0 (0.0)	9 (23.7)	
HB,g/L	110.28 ± 19.54	94.83 ± 17.09	<0.001
ALB, g/L	32.99 ± 4.15	32.36 ± 7.07	0.512
LOS, d	9.50 (8.00–12.25)	22.50 (15.00–34.50)	<0.001
ICU stay, h	0.00 (0.00–0.00)	106.50 (0.00–308.00)	<0.001

Data are presented as mean ± SD or median (IQR) for continuous variables, and n (%) for categorical variables. *p* values were obtained using Fisher’s exact test, Student’s t test, or Mann–Whitney U test, as appropriate. *p* < 0.05 was considered statistically significant. TSC, two-step clustering; NAOG, non-adverse outcome group; AOG, adverse outcome group; IAI, intra-abdominal infection; AL, anastomotic leakage; HB, hemoglobin; ALB, albumin; LOS, length of hospital stay; ICU, intensive care unit.

### 3.2 LASSO-based variable selection and comparison of clinical characteristics

**Figure 2:**
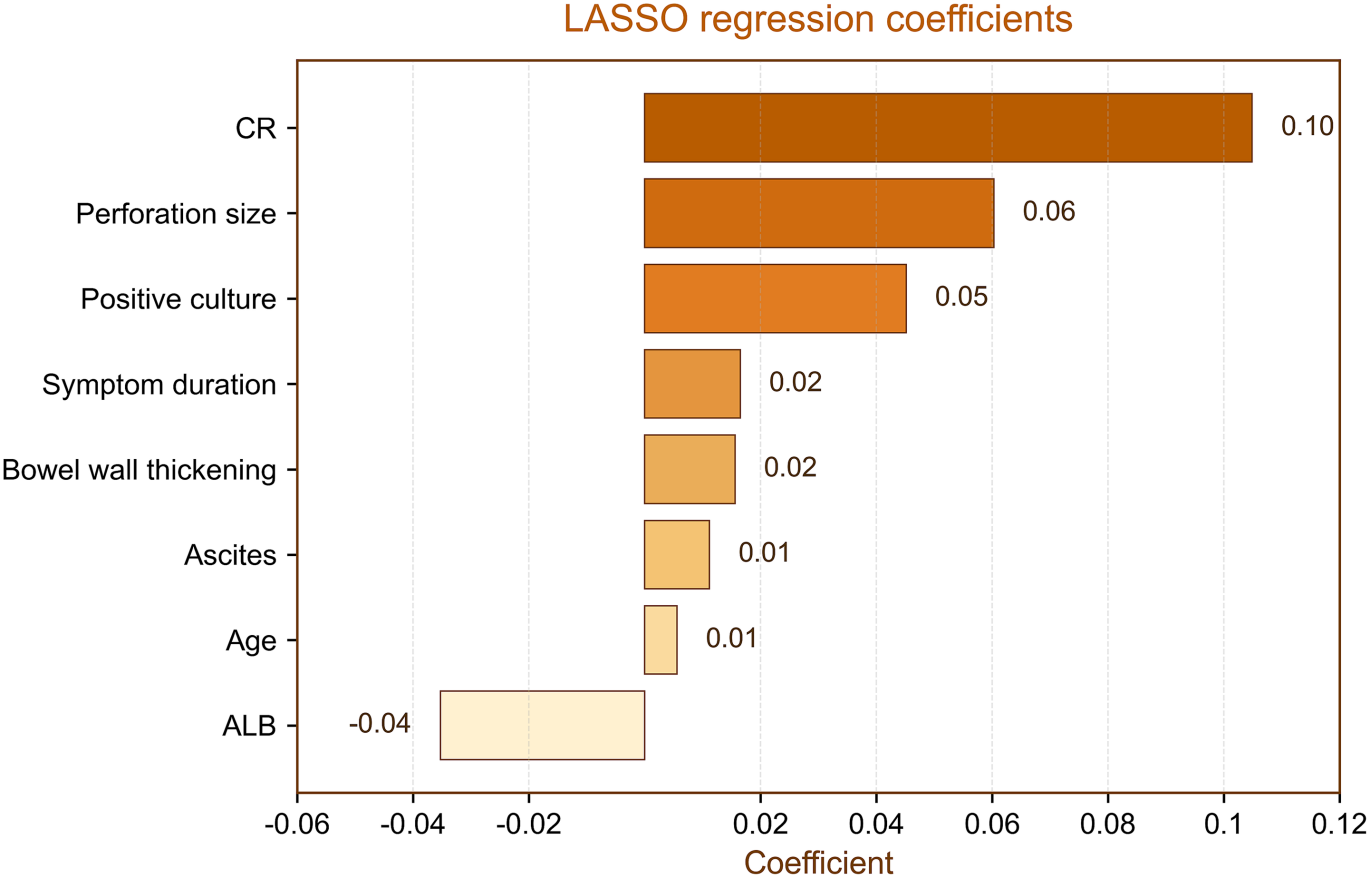
LASSO-based selection of candidate adjustment variables. LASSO, least absolute shrinkage and selection operator; CR, creatinine; ALB, albumin.

The variables retained by LASSO regression were as follows (Table 2; Figure 2): serum creatinine level (AOG: 130.50 [85.00–192.50] vs. NAOG: 83.50 [64.00–107.00]; *p* < 0.001), size of perforation (10.00 [7.25–15.00] vs. 8.00 [5.00–10.00]; *p* < 0.001), positive intraoperative culture (50.0% vs. 21.2%; *p* = 0.001), serum albumin level (32.54 ± 5.21 vs. 36.24 ± 6.53; *p* < 0.001), symptom duration (60.5% vs. 33.7%; *p* = 0.006), intestinal wall thickening (34.2% vs. 14.4%; *p* = 0.016), massive intra-abdominal effusion (36.8% vs. 18.3%; *p* = 0.026), and age (74.50 [70.00–80.75] vs. 69.50 [66.00–76.25]; *p* = 0.033).

**Table 2:** Comparison of clinical and surgical characteristics between NAOG and AOG.

Variable	NAOG (*n* = 104)	AOG (*n* = 38)	*p*-value
Baseline characteristics, n (%)			
Sex			0.831
Male	77 (74.0)	27 (71.1)	
Female	27 (26.0)	11 (28.9)	
Age years	69.50 (66.00–76.25)	74.50 (70.00–80.75)	0.033
Symptom duration			0.006
≤24 h	69 (66.3)	15 (39.5)	
>24 h	35 (33.7)	23 (60.5)	
Associated symptoms			0.846
No	42 (40.4)	14 (36.8)	
Yes	62 (59.6)	24 (63.2)	
NSAID history			0.334
No	96 (92.3)	33 (86.8)	
Yes	8 (7.7)	5 (13.2)	
Previous abdominal surgery			0.275
No	81 (77.9)	26 (68.4)	
Yes	23 (22.1)	12 (31.6)	
Comorbidity			0.182
No	50 (48.1)	13 (34.2)	
Yes	54 (51.9)	25 (65.8)	
Smoking history			0.844
No	68 (65.4)	24 (63.2)	
Yes	36 (34.6)	14 (36.8)	
Alcohol use history			0.765
No	93 (89.4)	33 (86.8)	
Yes	11 (10.6)	5 (13.2)	
ASA			0.326
Non-severe	41 (39.4)	11 (28.9)	
Severe	63 (60.6)	27 (71.1)	
Vital signs			
SBP mmHg	132.29 ± 19.40	125.00 ± 20.67	0.063
Temperature °C	36.60 (36.50–36.80)	36.75 (36.50–36.90)	0.296
Heart rate bpm	88.00 (80.75–100.25)	94.00 (81.75–107.50)	0.180
Laboratory findings			
HB g/L	133.62 ± 26.54	125.87 ± 34.18	0.211
PLT 10^9/L	252.41 ± 91.42	253.74 ± 122.89	0.952
ALB g/L	36.24 ± 6.53	32.54 ± 5.21	<0.001
K mmol/L	4.09 ± 0.59	4.35 ± 0.62	0.028
NA mmol/L	137.25 ± 4.13	136.73 ± 3.90	0.488
CL mmol/L	101.36 ± 4.94	102.29 ± 4.71	0.307
HCO mmol/L	22.40 ± 3.93	20.38 ± 3.80	0.007
CA mmol/L	2.22 ± 0.17	2.16 ± 0.16	0.061
WBC 10^9/L	11.50 (7.08–14.27)	10.15 (5.19–14.24)	0.680
NE 10^9/L	9.87 (5.82–13.23)	9.27 (4.38–13.15)	0.697
LN 10^9/L	0.57 (0.42–0.87)	0.61 (0.39–0.95)	0.800
ALT U/L	17.00 (13.00–26.00)	16.50 (12.00–30.75)	0.908
AST U/L	24.00 (19.75–32.25)	26.50 (19.50–36.75)	0.349
CR μmol/L	83.50 (64.00–107.00)	130.50(85.00–192.50)	<0.001
GLU mmol/L	7.51 (5.86–9.44)	7.51 (6.40–9.95)	0.599
PT s	13.50 (12.70–14.62)	15.50 (14.15–16.68)	<0.001
APTT s	33.85 (30.68–38.66)	38.15 (34.47–43.80)	<0.001
FIB g/L	4.44 (3.74–6.25)	7.11 (4.23–14.88)	0.025
TT s	16.20 (14.50–17.00)	15.45 (7.49–16.70)	0.181
D-D mg/L	2.33 (1.40–3.70)	4.08 (2.61–5.34)	<0.001
Preoperative imaging, n (%)			
Multifocal free air			0.133
No	59 (56.7)	16 (42.1)	
Yes	45 (43.3)	22 (57.9)	
Bowel wall thickening			0.016
No	89 (85.6)	25 (65.8)	
Yes	15 (14.4)	13 (34.2)	
Intraoperative findings, n (%)			
Perforation size mm	8.00 (5.00–10.00)	10.00 (7.25–15.00)	<0.001
Positive culture			0.001
No	82 (78.8)	19 (50.0)	
Yes	22 (21.2)	19 (50.0)	
Ascites			0.026
≤1,000 ml	85 (81.7)	24 (63.2)	
>1,000 ml	19 (18.3)	14 (36.8)	
Surgical approach			0.072
Open	31 (29.8)	18 (47.4)	
Laparoscopic	73 (70.2)	20 (52.6)	
Perforation site			1.000
Stomach	67 (64.4)	25 (65.8)	
Duodenum	37 (35.6)	13 (34.2)	

Data are presented as mean ± SD or median (IQR) for continuous variables, and n (%) for categorical variables. *p* values were obtained using Fisher’s exact test, Student’s t test, or Mann–Whitney U test, as appropriate. *p* < 0.05 was considered statistically significant. NAOG, non-adverse outcome group; AOG, adverse outcome group; ASA, American Society of Anesthesiologists; SBP, systolic blood pressure; HB, hemoglobin; PLT, platelet count; ALB, albumin; K, potassium; Na, sodium; Cl, chloride; HCO, bicarbonate; CA, calcium; WBC, white blood cell count; NE, neutrophil count; LN, lymphocyte count; ALT, alanine aminotransferase; AST, aspartate aminotransferase; CR, creatinine; GLU, glucose; PT, prothrombin time; APTT, activated partial thromboplastin time; FIB, fibrinogen; TT, thrombin time; D-D, D-dimer.

### 3.3 Construction and performance of the primary DML model

In the primary analysis, each 1-day delay in postoperative EN initiation was associated with a 3.55% increase in the risk of adverse outcome (95% CI: 1.41–6.61%). The ATE was consistently positive across models, with estimated effect sizes ranging from 2.74 to 5.22% per day. The substantial overlap of the 95% CIs across models suggested overall consistency. Stability analysis showed that the standard deviation of the ATE across five-fold cross-fitting ranged from 1.28 to 2.72. Among the evaluated models, SVM had the lowest SD across folds (1.28), indicating relatively low estimation variability. As the primary model, LR showed an SD across folds of 1.77, second only to SVM, and also demonstrated good stability (Table 3).

**Table 3:** DML results with different first-stage models.

Model	ATE(%/day)	95% CI(%/day)	SD across folds
LR	3.55	1.41–6.61	1.77
RF	4.03	1.89–7.62	1.80
XGB	2.74	0.67–6.04	2.30
SVM	4.33	2.04–8.51	1.28
MLP	5.22	2.61–9.41	2.72

ATE represents the percentage change in adverse outcome risk associated with each 1-day delay in postoperative EN initiation. SD across folds refers to the standard deviation of ATE estimates across five-fold cross-fitting and was used to assess model stability. DML, double machine learning; ATE, average treatment effect; CI, confidence interval; SD, standard deviation; EN, enteral nutrition; LR, linear regression; RF, random forest; SVM, support vector machine; MLP, multilayer perceptron; XGB, extreme gradient boosting.

### 3.4 Impact of delayed EN initiation on adverse outcome risk

**Figure 3:**
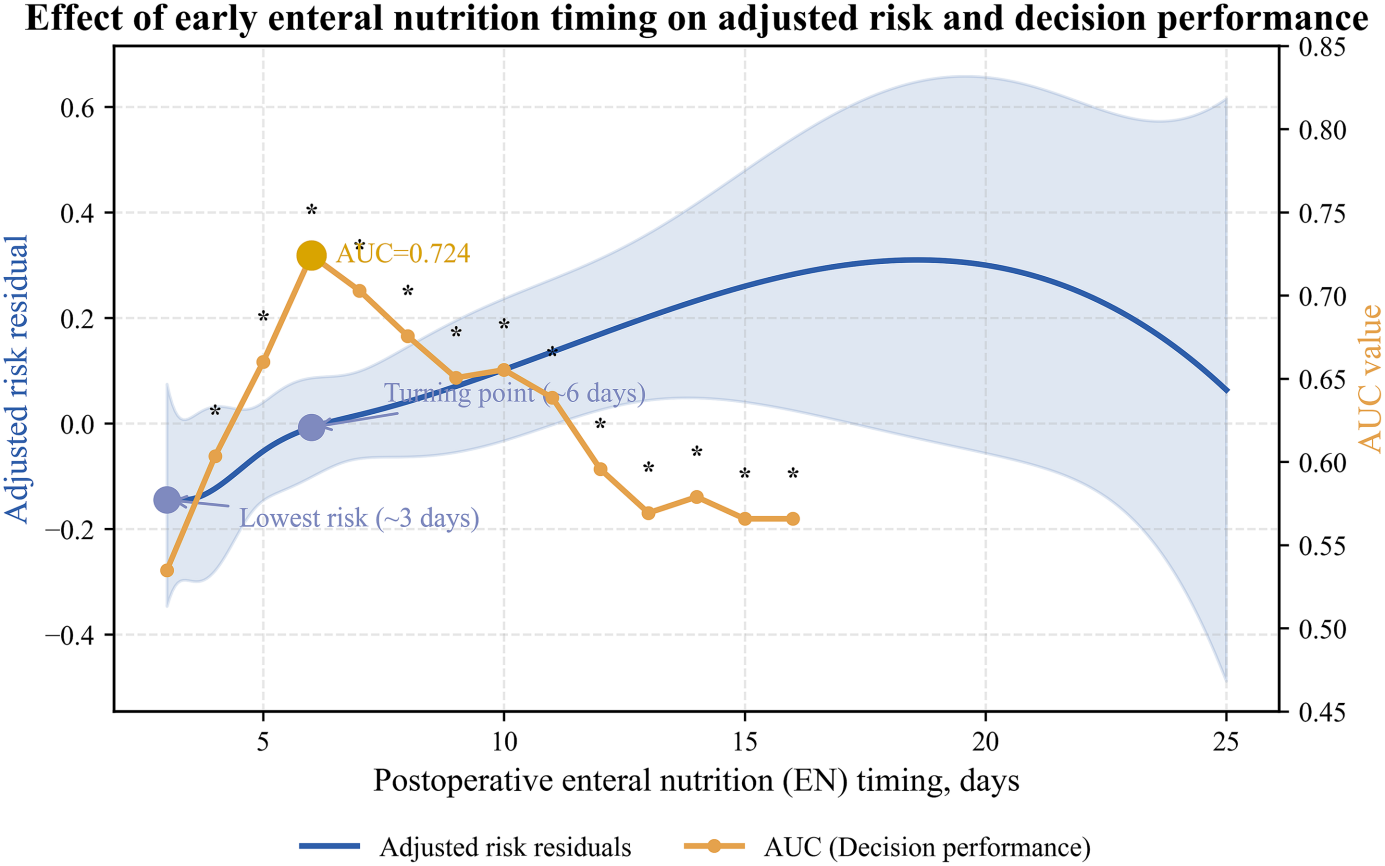
Effect of postoperative enteral nutrition timing on residualized risk and model performance. The figure shows the relationship between postoperative EN initiation time, residual risk, and model performance in the primary analysis (linear regression for the treatment model and logistic regression for the outcome model). The blue curve represents the spline-smoothed residual risk derived from the primary DML model, with the shaded area indicating the 95% confidence interval. The orange line, referenced to the right *y*-axis, shows the AUC from the single-level decision-tree analysis across different EN initiation thresholds. The residual risk was lowest within 3 postoperative days and returned to zero around day 6. Using day 6 as the threshold, the decision-tree model achieved the highest discriminative performance, with an AUC of 0.724. EN, enteral nutrition; DML, double machine learning; AUC, area under the receiver operating characteristic curve; CI, confidence interval.

Using spline regression analysis, the relationship between the timing of postoperative EN initiation and the risk of adverse outcomes was evaluated (Figure 3). The results demonstrated a nonlinear association between EN initiation timing and risk. The lowest risk point was observed on postoperative day 3, after which the risk gradually increased. When patients were grouped by postoperative day 4 as the cutoff, a significant difference in the incidence of adverse outcomes was observed between the two groups (*p* < 0.05). On day 6, spline regression showed that the risk residual returned to zero, while the decision tree model identified 6 days as the optimal threshold, providing the highest discriminatory power for predicting adverse outcomes (AUC = 0.724).

## 4 Discussion

EN is a key component of postoperative recovery, and its timing plays a pivotal role in maintaining mucosal integrity, controlling infection, and improving overall outcomes (31, 32). Although early EN has demonstrated clear benefits in various surgical settings, evidence among ePPUs remains limited (33). These patientspresent with limited nutritional reserves and diminished physiological tolerance, making the timing of EN particularly challenging (34). Therefore, defining the optimal timing of early EN in ePPUs may help improve postoperative outcomes.

This study employed TSC analysis based on multiple postoperative outcome indicators, enabling the simultaneous processing of both continuous and categorical variables (35). Compared with the use of a single outcome indicator, this approach offers a more comprehensive representation of patients’ multidimensional prognostic profiles. During intraoperative source control, complete eradication of infection is often challenging because bacteria adhere firmly to the peritoneal mesothelial surface, rendering surgical irrigation insufficient to eliminate the microbial load (3, 36). For ePPUs, the compromised immunity and residual infection frequently induced scattered or deep-seated abscesses that are difficult to drain effectively, resulting in uncontrolled abdominal infection (27, 33, 37, 38). Most intra-abdominal infections are caused by gram-negative enteric bacteria, whose endotoxins are highly pathogenic (39). These endotoxins, together with other inflammatory cytokines, can be readily absorbed into the systemic circulation through the peritoneum’s extensive vascular and lymphatic networks, predisposing ePPUs to septic shock and progressive multiple organ dysfunction (36, 40). Diminished immune defense capacity increases the susceptibility to inflammation, rendering them more prone to developing systemic inflammatory response syndrome (SIRS) (41, 42). This condition further enhances vascular permeability, impairs microcirculatory function, and aggravates distal organ injury, ultimately leading to prolonged ICU stays, extended LOS, and a higher incidence of complications (16, 43).

During the LASSO feature selection process, serum creatinine exhibited the greatest contribution to outcome prediction. Extensive intra-abdominal contamination induces a systemic inflammatory response, whereas perforation and capillary leakage cause fluid loss into the third space. In conjunction with vomiting, fasting, and fever, these factors lead to systemic hypoperfusion and subsequent acute kidney injury (AKI) (40, 42). Multicenter and prospective studies have consistently demonstrated a significant association between AKI and 30-day mortality as well as postoperative complications, with elevated serum creatinine serving as an independent predictor of both (44, 45). Moreover, delayed disease course, larger perforation size, and positive intraoperative cultures have been identified as independent risk factors, indicating a prolonged and more extensive course of IAI with increased contamination burden—all of which intensify inflammation and aggravate AKI (3, 36, 37). Therefore, serum creatinine also represents a high-risk marker reflecting the severity of perforation.

At our center, the timing of EN initiation was primarily determined by the attending surgeon based on the patient’s overall clinical condition, with particular consideration given to hemodynamic stability, the presence of persistent shock or marked circulatory instability, recovery of gastrointestinal function, and readiness for oral intake. Notably, the clinical variables ultimately retained by LASSO regression also represent important factors influencing the decision to delay EN. Elevated serum creatinine often indicates inadequate renal perfusion and reflects ongoing hemodynamic instability. Larger perforation size usually suggests more severe local tissue injury and greater tension at the repair site, while low albumin may further contribute to tissue edema and impaired healing capacity at the repair site. Prolonged symptom duration generally reflects a longer-lasting infectious process and a greater inflammatory burden. In addition, older age is often associated with poorer vascular status and impaired microcirculatory perfusion. Together, these factors may delay gastrointestinal recovery and thereby affect the timing of EN initiation. However, because of the retrospective design of this study, the specific clinical reasons for delaying EN were not systematically recorded for each patient. Prospective studies are therefore needed to more comprehensively document and control indications for EN initiation, reasons for delay, and related management factors.

Spline-based risk analysis further suggested that overall risk increased cumulatively with delayed EN initiation, broadly consistent with the general consensus that earlier EN confers greater clinical benefit. Early EN enhances intestinal blood flow through feeding stimulation, promotes epithelial regeneration, strengthens tight junction protein synthesis, and supports the proliferation of beneficial flora, thereby reducing the translocation of pathogenic bacteria. It may also upregulate anti-inflammatory cytokines and mitigate systemic inflammatory responses (32, 46, 47). Existing evidence in upper gastrointestinal surgery and critically ill populations generally indicates that, provided hemodynamic stability and gastrointestinal tolerance are achieved, avoiding unnecessary delay in EN is associated with better recovery outcomes. Jeong et al. reported that early feeding after gastrectomy in elderly patients requires close monitoring (18). The ESPEN guidelines recommend resuming oral intake as early as possible after surgery, while also emphasizing cautious advancement according to individual tolerance in older patients (12). Reviews in critically ill populations have likewise suggested that early EN may reduce infection-related complications, although feeding intolerance is not uncommon in high-risk patients and decisions should therefore be individualized according to gastrointestinal tolerance, aspiration risk, and circulatory status, with strategies such as jejunal feeding or continuous infusion considered when necessary (32, 43, 48). For ePPUs, however, direct evidence remains limited. In clinical practice, impaired tissue repair capacity and severe IAI often lead clinicians to defer EN initiation, particularly in the absence of appropriate feeding strategies to offset these risks (14, 34).

When EN initiation is further delayed, prolonged fasting may damage the intestinal barrier, promote the translocation of bacterial products and endotoxins, and subsequently trigger systemic inflammation (13, 32, 47). The resulting inflammatory response may further injure epithelial cells and tight junctions, aggravate barrier dysfunction, impair nutrient absorption, and ultimately worsen nutritional status (13). In our analysis, spline regression showed that when EN initiation was delayed beyond postoperative day 6, the residual risk shifted from negative to positive, indicating a transition to a higher-risk level. This finding suggests that when EN initiation was delayed beyond postoperative day 6, its protective effect may have been progressively offset by cumulative inflammatory injury. Consistently, the decision tree model showed the best discriminatory performance for adverse outcomes when postoperative day 6 was used as the cutoff. Together, these findings indicate a clinically relevant risk transition around postoperative day 6.

To facilitate earlier EN in patients at high risk for conventional oral or gastric feeding, alternative distal feeding strategies may be needed. EN via the jejunal route is essential for maintaining nutritional support and preserving the intestinal barrier in patients with upper gastrointestinal perforation or recent anastomoses (49). However, intraoperative placement is often challenging due to the limited laparoscopic workspace, and early postoperative endoscopic insertion may increase mechanical irritation and gastric insufflation, thereby elevating the risk of anastomotic leakage. To overcome these limitations, fluoroscopic (digital subtraction angiography, DSA)–guided, guidewire-assisted jejunal tube placement provides a safe and precise alternative, as demonstrated in Supplementary Videos 1–4 (50). Under real-time fluoroscopic monitoring, the tube is advanced beyond the duodenum or the ligament of Treitz, minimizing contact with the anastomosis and preventing excessive gastric insufflation. Previous studies have reported technical success rates of 92–100% with minimal complications (51). At our center, this approach may enable early distal feeding while reducing local irritation, the risk of anastomotic leakage, and inflammation associated with delayed EN, thereby offering a potential new clinical strategy for establishing early EN in high-risk elderly patients.

This study has several limitations. First, the sample size was limited and derived from a single center, which may have reduced statistical power. In addition, the retrospective design could not account for all unmeasured confounders, such as surgeon experience, intraoperative hemodynamic fluctuations, and patient or family preferences in clinical decision-making. Therefore, the findings should be interpreted as preliminary exploratory evidence and require further validation in larger samples and prospective studies. Finally, external validation was not performed, and the generalizability of the model remains to be evaluated in independent cohorts. Future large-scale, prospective, multicenter studies are needed to confirm the robustness of our findings and to further optimize postoperative nutritional strategies for elderly patients with perforated peptic ulcer.

## 5 Conclusion

Earlier initiation of EN was associated with progressively lower postoperative risk, with each one-day delay increasing the probability of adverse outcomes by approximately 4%. Postoperative day 6 represented the critical threshold beyond which the protective effect of EN markedly declined.

## Data Availability

The datasets generated and/or analyzed during the current study are available from the corresponding author upon reasonable request, subject to institutional and ethical restrictions.

## Supplementary material

Supplementary Videos 1–4 Demonstrate the technique of jejunal feeding tube placement used to facilitate early distal enteral nutrition in high-risk patients.
